# Supplementary material for: Sexual Dimorphism in Body Weight Loss, Improvements in Cardiometabolic Risk Factors and Maintenance of Beneficial Effects 6 Months after a Low-Calorie Diet: Results from the Randomized Controlled DiOGenes Trial
Source: Nutrients. 2021 May 10;13(5):1588. doi: 10.3390/nu13051588 (PMC8151806; doi:10.3390/nu13051588)
Supplement: Supplementary file 1 [file nutrients-13-01588-s001.zip › nutrients-1182766-supplementary.pdf]

Table S1. Standardized beta's of the link between sex and the changes in individual lipid species following loss and weight maintenance

	Change from CID1-2		Change from CID2-3	
Lipid species	Std. beta ± SE	*q*-value	Std. beta ± SE	*q*-value
TAG (46:2)	-0.023 ± 0.018	0.290	0.023 ± 0.044	0.599
TAG (48:0)	-0.038 ± 0.021	0.159	-0.070 ± 0.047	0.154
TAG (48:1)	-0.010 ± 0.022	0.764	-0.099 ± 0.046	0.041
TAG (48:2)	-0.004 ± 0.023	0.910	-0.072 ± 0.044	0.114
TAG (48:3)	-0.002 ± 0.024	0.950	-0.024 ± 0.041	0.570
TAG (48:4)	-0.057 ± 0.026	0.078	0.045 ± 0.042	0.299
TAG (50:0)	-0.038 ± 0.022	0.169	-0.140 ± 0.047	0.005
TAG (50:1)	0.009 ± 0.028	0.831	-0.119 ± 0.046	0.015
TAG (50:2)	0.070 ± 0.029	0.046	-0.132 ± 0.045	0.005
TAG (50:3)	0.068 ± 0.030	0.061	-0.099 ± 0.040	0.020
TAG (50:4)	0.047 ± 0.029	0.182	-0.075 ± 0.038	0.065
TAG (50:5)	0.031 ± 0.025	0.295	-0.056 ± 0.041	0.182
TAG (51:1)	-0.005 ± 0.024	0.874	-0.160 ± 0.045	<0.001
TAG (51:2)	-0.008 ± 0.026	0.831	-0.070 ± 0.043	0.119
TAG (51:3)	0.042 ± 0.031	0.255	-0.108 ± 0.037	0.006
TAG (51:4)	0.046 ± 0.030	0.220	-0.097 ± 0.038	0.016
TAG (52:0)	-0.037 ± 0.017	0.068	-0.210 ± 0.047	<0.001
TAG (52:1)	-0.001 ± 0.025	0.958	-0.177 ± 0.046	<0.001
TAG (52:2)	0.065 ± 0.036	0.158	-0.135 ± 0.043	0.004
TAG (52:3)	0.105 ± 0.034	0.010	-0.098 ± 0.041	0.024
TAG (52:4)	0.118 ± 0.034	<0.001	-0.094 ± 0.040	0.024
TAG (52:5)	0.067 ± 0.027	0.0428	-0.098 ± 0.039	0.017
TAG (52:6)	0.037 ± 0.022	0.174	-0.110 ± 0.040	0.011
TAG (53:1)	-0.037 ± 0.027	0.253	-0.053 ± 0.045	0.259
TAG (54:0)	0.014 ± 0.009	0.215	0.072 ± 0.044	0.118
TAG (54:0)	-0.034 ± 0.023	0.228	-0.151 ± 0.041	<0.001
TAG (54:1)	-0.025 ± 0.018	0.248	-0.235 ± 0.047	<0.001
TAG (54:2)	0.043 ± 0.031	0.248	-0.193 ± 0.043	<0.001
TAG (54:3)	0.050 ± 0.036	0.248	-0.126 ± 0.041	0.004
TAG (54:4)	0.065 ± 0.034	0.136	-0.115 ± 0.039	0.006
TAG (54:5)	0.094 ± 0.032	0.018	-0.147 ± 0.040	<0.001
TAG (54:6)	0.078 ± 0.029	0.024	-0.152 ± 0.041	<0.001
TAG (54:7)	0.045 ± 0.026	0.163	-0.158 ± 0.042	<0.001
TAG (55:1)	-0.029 ± 0.026	0.344	-0.184 ± 0.047	<0.001
TAG (55:2)	0.011 ± 0.035	0.831	-0.077 ± 0.043	0.094
TAG (55:3)	0.064 ± 0.035	0.158	-0.076 ± 0.043	0.094
TAG (56:1)	-0.057 ± 0.013	<0.001	-0.215 ± 0.047	<0.001
TAG (56:2)	-0.043 ± 0.015	0.024	-0.251 ± 0.048	<0.001
TAG (56:3)	-0.014 ± 0.028	0.760	-0.198 ± 0.043	<0.001
TAG (56:4)	0.014 ± 0.030	0.764	-0.090 ± 0.040	0.033
TAG (56:5)	0.028 ± 0.032	0.478	-0.108 ± 0.037	0.006
TAG (56:6)	0.105 ± 0.032	0.007	-0.139 ± 0.040	<0.001
TAG (56:7)	0.077 ± 0.030	0.029	-0.177 ± 0.043	<0.001
TAG (56:8)	0.093 ± 0.030	0.010	-0.184 ± 0.047	<0.001
TAG (57:1)	-0.029 ± 0.025	0.317	-0.216 ± 0.045	<0.001
TAG (57:2)	0.014 ± 0.033	0.786	-0.198 ± 0.042	<0.001
TAG (58:1)	-0.057 ± 0.015	<0.001	-0.184 ± 0.049	<0.001
TAG (58:2)	-0.059 ± 0.014	<0.001	-0.185 ± 0.049	<0.001
TAG (58:3)	-0.045 ± 0.014	0.009	-0.198 ± 0.049	<0.001
TAG (58:6)	0.080 ± 0.030	0.024	-0.131 ± 0.040	0.002
TAG (58:8)	0.086 ± 0.031	0.024	-0.156 ± 0.046	0.002

TAG (58:9)	0.103 ± 0.031	0.007	-0.175 ± 0.045	<0.001
TAG (58:10)	0.098 ± 0.031	0.010	-0.149 ± 0.047	0.004
TAG (59:0)	-0.031 ± 0.023	0.258	-0.123 ± 0.040	0.004
TAG (60:2)	-0.056 ± 0.013	<0.001	-0.197 ± 0.050	<0.001
DAG (36:2)			-0.139 ± 0.039	<0.001
DAG (36:3)			-0.082 ± 0.035	0.020
CholE02	0.038 ± 0.033	0.250		
CholE05	0.208 ± 0.039	<0.001		
CholE06	0.208 ± 0.039	<0.001		
LPC (14:0)	-0.066 ± 0.033	0.069	0.031 ± 0.041	0.460
LPC (16:0)	-0.072 ± 0.039	0.081	-0,178 ± 0.041	<0.002
LPC (16:1)	-0.010 ± 0.034	0.776	-0.061 ± 0.043	0.176
LPC (18:0)	-0.042 ± 0.039	0.308	-0.102 ± 0.040	0.017
LPC (18:1)	-0.275 ± 0.037	<0.001	-0.159 ± 0.047	0.002
LPC (18:2)	-0.235 ± 0.040	<0.001	-0.146 ± 0.040	<0.001
LPC (18:3)	-0.171 ± 0.029	<0.001	-0.097 ± 0.043	0.032
LPC (20:3)	-0.198 ± 0.036	<0.001	-0.108 ± 0.042	0.016
LPC (20:4)	-0.127 ± 0.040	0.003	-0.180 ± 0.044	<0.001
LPC (20:5)	-0.138 ± 0.033	<0.001	-0.194 ± 0.048	<0.001
LPC (22:6)	-0.198 ± 0.038	<0.001	-0.142 ± 0.047	0.005
LPCO (16:1)	-0.177 ± 0.039	<0.001		
LPCO (18:1)	-0.068 ± 0.039	0.085		
SM (d18:1/14:0)	0.094 ± 0.036	0.018	0.092 ± 0.038	0.027
SM (d18:1/15:0)	0.140 ± 0.041	0.003	0.091 ± 0.039	0.031
SM (d18:1/16:0)	0.103 ± 0.040	0.018	0.074 ± 0.037	0.059
SM (d18:1/16:1)	0.185 ± 0.044	<0.001	0.122 ± 0.039	0.007
SM (d18:1/17:0)	0.201 ± 0.040	<0.001	0.027 ± 0.037	0.528
SM (d18:1/18:0)	0.284 ± 0.040	<0.001	0.045 ± 0.030	0.162
SM (d18:1/18:1)	0.255 ± 0.041	<0.001	0.070 ± 0.031	0.033
SM (d18:1/18:2)	0.289 ± 0.040	<0.001	0.097 ± 0.037	0.016
SM (d18:1/20:0)	-0.054 ± 0.033	0.147	0.104 ± 0.036	0.009
SM (d18:1/20:1)	0.008 ± 0.040	0.888	0.144 ± 0.040	<0.001
SM (d18:1/21:0)	-0.028 ± 0.033	0.467	0.126 ± 0.037	0.004
SM (d18:1/22:0)	-0.049 ± 0.035	0.211	0.143 ± 0.040	<0.001
SM (d18:1/22:1)	-0.005 ± 0.038	0.898	0.217 ± 0.040	<0.001
SM (d18:1/23:0)	-0.041 ± 0.034	0.271	0.108 ± 0.036	0.008
SM (d18:1/23:1)	0.017 ± 0.038	0.724	0.152 ± 0.041	<0.002
SM (d18:1/24:0)	-0.073 ± 0.034	0.052	0.115 ± 0.041	0.012
SM (d18:1/24:1)	0.111 ± 0.041	0.016	0.082 ± 0.036	0.031
SM (d18:1/24:2)	0.150 ± 0.041	<0.001	0.112 ± 0.038	0.008
SM (d18:1/25:0)	0.054 ± 0.033	0.147	0.024 ± 0.039	0.574
SM (d18:1/25:1)	0.197 ± 0.041	<0.001	-0.016 ± 0.038	0.677

Standardized beta ± standard error in linear mixed model with the individual lipid species as dependent variable, sex as fixed effect and study center as random effect. Adjusted for age, body weight at baseline, weight loss and baseline value of the dependent variable in the weight loss phase (from CID1 to CID2) and adjusted for age, baseline weight, baseline value of the dependent variable, change of the dependent variable during weight loss, weight change during the weight loss phase and weight maintenance phase and diet in the weight maintenance phase (from CID2 to CID3). *q*-value = False Discovery Rate adjusted *p*-value. Individual lipid species only shown when the change in total lipid group revealed statistically significant association with sex (Figure 2).
